# *In Vivo* Healthy Knee Kinematics during Dynamic Full Flexion

**DOI:** 10.1155/2013/717546

**Published:** 2012-12-23

**Authors:** Satoshi Hamai, Taka-aki Moro-oka, Nicholas J. Dunbar, Hiromasa Miura, Yukihide Iwamoto, Scott A. Banks

**Affiliations:** ^1^Department of Mechanical and Aerospace Engineering, University of Florida, 231 MAE-A Building, P.O. Box 116250, Gainesville, FL 32611-6250, USA; ^2^Department of Orthopaedic Surgery, Faculty of Medical Sciences, Kyushu University, 3-1-1 Maidashi, Higashi-ku, Fukuoka 812-8582, Japan; ^3^Department of Orthopaedic Surgery, Faculty of Medicine, Ehime University, 10-13 Dogo-himata, Matsuyama, Ehime 790-8577, Japan

## Abstract

Healthy knee kinematics during dynamic full flexion were evaluated using 3D-to-2D model registration techniques. Continuous knee motions were recorded during full flexion in a lunge from 85° to 150°. Medial and lateral tibiofemoral contacts and femoral internal-external and varus-valgus rotations were analyzed as a function of knee flexion angle. The medial tibiofemoral contact translated anteroposteriorly, but remained on the center of the medial compartment. On the other hand, the lateral tibiofemoral contact translated posteriorly to the edge of the tibial surface at 150° flexion. The femur exhibited external and valgus rotation relative to the tibia over the entire activity and reached 30° external and 5° valgus rotations at 150° flexion. Kinematics' data during dynamic full flexion may provide important insight as to the designing of high-flexion total knee prostheses.

## 1. Introduction

3D-to-2D model registration techniques [1–4] and MRI-based methods [5–9] are used for assessing 3D knee kinematics without implants. Although deep knee flexion is an important function for many activities of daily living, few studies [4, 7, 9] have explored the *in vivo* knee kinematics beyond 140° of flexion. Kinematic analysis of healthy knees during dynamic full flexion is one key to designing for full flexion after total knee arthroplasty. 

The purpose of this study was to observe healthy kinematics during full flexion in a lunge using 3D-to-2D model registration techniques. We sought to answer a specific question: How does the femur translate and rotate relative to the tibia during dynamic full flexion? 

## 2. Materials and Methods 

Five healthy male subjects, averaging 29 years (28–30), 172 cm (169–177), and 68 kg (55–80), gave informed consent to participate in this Institutional Review Board approved study. Continuous knee motions were recorded using a flat panel detector (Hitachi, Clavis, Tokyo, Japan: 3 frames/sec, 0.20 × 0.20 mm/pixel resolution) during full flexion in a lunge with their foot placed on a 25-cm step. 

**Figure 1:**
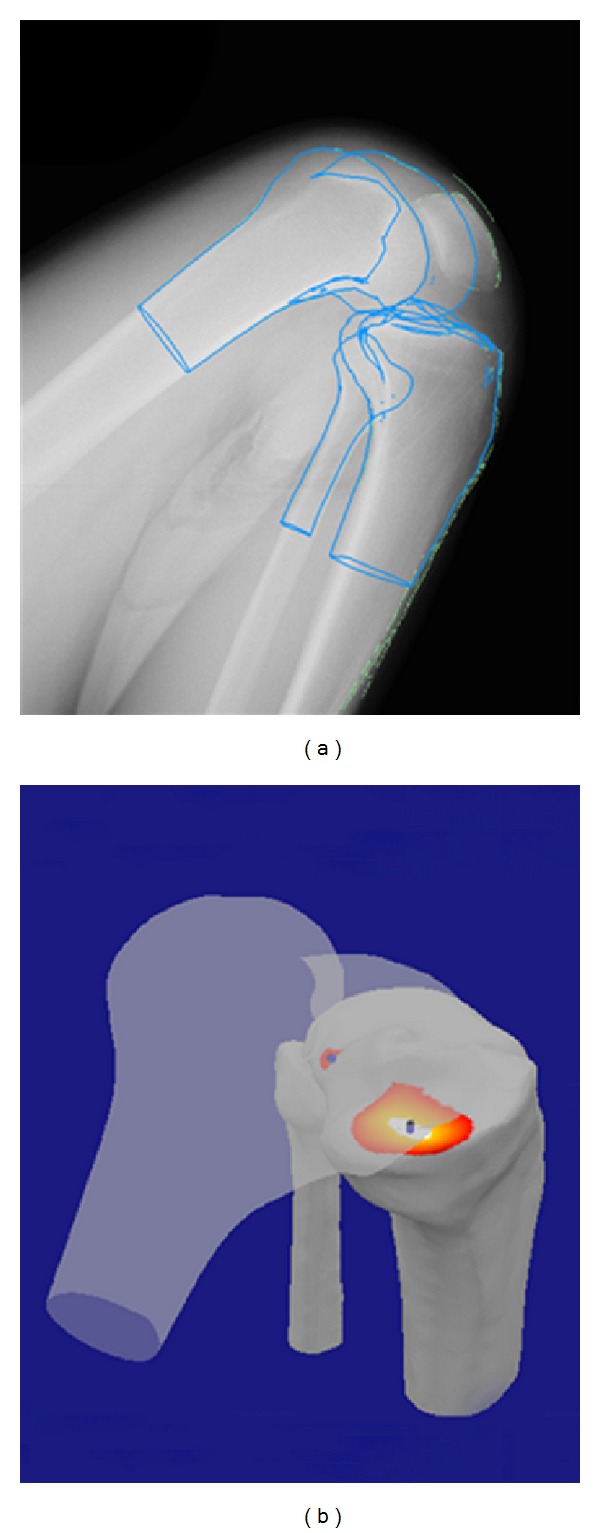
3D-to-2D CT model-to-flat panel image registration techniques were used to determine the *in vivo* healthy knee kinematics (a). Medial and lateral tibiofemoral contacts were computed as the geometric center of the region having less than 6 mm tibiofemoral separation (b).

Cortical bone edges were segmented from CT images (Aquilion, Toshiba, Tochigi, Japan) using commercial software (SliceOmatic, Tomovision, Montreal, CA, USA), and these point clouds were converted into polygonal surface models (Geomagic Studio, Raindrop Geomagic, NC, USA). Bone model-embedded Cartesian coordinate systems for each femur and tibia were aligned with the cylindrical axis described by Eckhoff et al. [10]. The 3D position and orientation of the femur and tibia/fibula were determined using 3D-to-2D CT model-to-flat panel image registration techniques [3, 4] (Figure 1(a)). Medial and lateral tibiofemoral contacts were computed as the geometric center of the region having less than 6 mm tibiofemoral separation [3, 4] (Figure 1(b)). Femoral internal-external and varus-valgus rotations relative to the tibia were analyzed as a function of knee flexion angle. The center of axial rotation was determined from the femoral flexion/extension axis [11, 12]. The mediolateral location of the center of rotation was normalized to the dimensions of each tibial plateau, and expressed as a percentage of the tibial width, −50% (lateral) to +50% (medial). Spline interpolation with 10° flexion increments was used to create average kinematics for the group. The best-case accuracy of this matching method was 0.53 mm for in-plane translation, 1.6 mm for out-of-plane translation, and 0.54° for rotations in a previous study [3].

## 3. Results and Discussion

**Figure 2:**
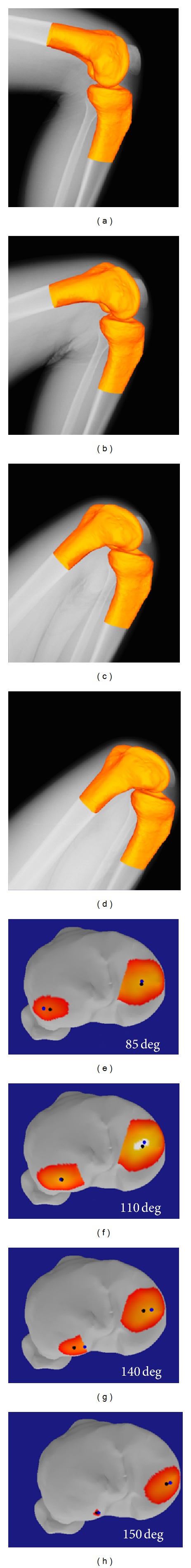
Matching of bone models to x-ray images: (a) 85°, (b) 110°, (c) 140°, and (d) 150° flexion. Medial and lateral tibiofemoral contacts were computed during weight-bearing dynamic knee flexion (e, f, g, and h). Black points on the tibial surfaces in the lower stand mean geometric center of the contact regions.

**Figure 3:**
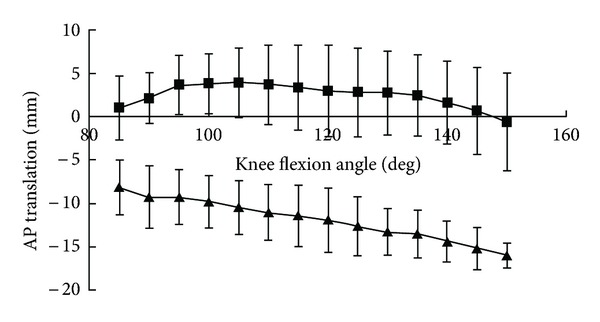
Anteroposterior (AP) translations of the medial (■) and lateral (▲) tibiofemoral contacts on the tibial surfaces.

**Figure 4:**
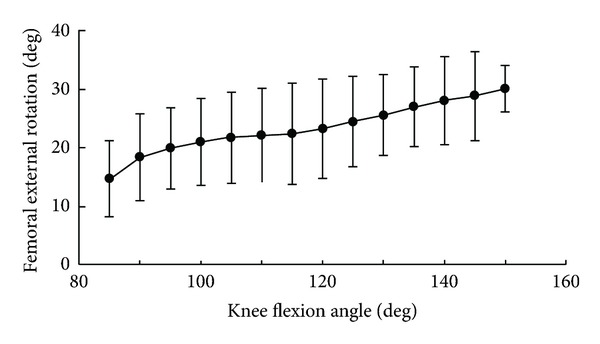
Femoral external rotation relative to the tibia.

**Figure 5:**
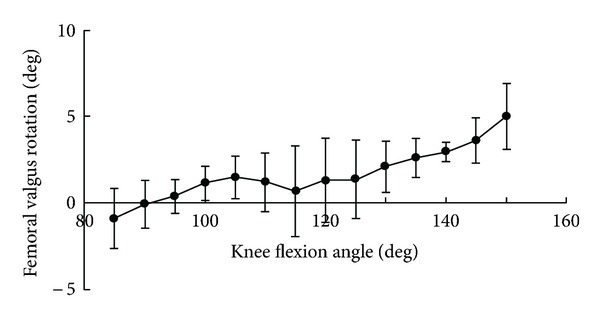
Femoral valgus rotation relative to the tibia.

The medial tibiofemoral contact translated anteroposteriorly, but remained centered in the medial compartment (Figure 2). The lateral tibiofemoral contact translated posteriorly to the edge of the tibial surface at 150° flexion. From 85° to 150° flexion, the medial and lateral tibiofemoral contacts demonstrated 2 mm and 8 mm posterior translations on the tibial surface, respectively (Figure 3). The lateral femoral condyle had larger posterior translations than the medial femoral condyle and femoral external rotation increased with knee flexion. The femur exhibited 15° external rotation relative to the tibia over the entire activity and reached 30° external rotation at 150° flexion (Figure 4). A medial center of rotation (+24%) was demonstrated for the lunge activity from 85° to 150° flexion. The femoral valgus rotation relative to the tibia was demonstrated over the entire activity and reached 5° at 150° flexion (Figure 5). 

In this study, 3D-to-2D model registration techniques have been used to measure healthy knee kinematics during dynamic full flexion. The medial contact remained centered on the tibial surface. On the contrary, the lateral contact translated posteriorly to the edge of the tibial surface at full flexion. The femur externally rotated relative to tibia around a medially positioned axis from 85° to 150° flexion and reached 30° external and 5° valgus rotation relative to the tibia at 150° flexion. 

Anteroposterior translations of the medial and lateral tibiofemoral contacts during deep knee flexion are generally consistent with previous 3D-to-2D model registration [3] and MR analyses [7, 9]. Nakagawa et al. [7] reported that the lateral condyle had the larger translation and lies posterior to the tibia at 162°. The lateral meniscus also moves with the lateral condyle, assuming a key role in distributing tibiofemoral compressive force [8]. Moro-oka et al. [3] reported that the medial contact translated 3 mm anteriorly and the lateral contact translated 8 mm posteriorly during kneeling from 100° to 150°. On the other hand, Pinskerova et al. [9] used the centers of the posterior circular portions of the medial and lateral femoral condyles (termed the Flexion Facet Centers) and reported that the medial condyle moved back 8 mm and the lateral 5 mm from 120° to 160°. Variations in the anteroposterior translations could be explained by different anatomic coordinate systems, activity, and foot position [3, 5, 13]. 

The average center of rotation provides a simple metric to describe the general pattern of knee motion over an entire activity [1]. This metric permits intuitive comparisons between activities. In this study, the center of axial rotation for lunging was approximately located at the center of the medial compartment, +24%. This is consistent with reports from other papers, which examined nonambulatory activities. Yamaguchi et al. [12] reported that average center of rotation for squatting from extension to 110° was located in the medial tibia, +23%. Komistek et al. [1] compared kinematics in healthy knees during deep knee bends and rising from a chair and showed a medial center of rotation for both activities. Johal et al. [6] also similarly showed a medial center of rotation pattern during several sagittal plane activities. Moro-oka et al. [3] reported femoral external rotation increased with knee flexion for stair from 0° to 70°, squat from 0° to 140°, and kneel from 100° to 150°. On the other hand, recent studies using motion capture and dynamic stereo X-ray imaging have reported greater medial than lateral translations during walking and running and suggest that there is a lateral center for tibial axial rotations during weight-bearing activities near extension [14–16]. 

The femur exhibited 5° valgus rotation at 150° flexion. Since the medial and lateral tibial surfaces have different sagittal slopes, valgus rotation can result from the condyles moving anteroposteriorly while remaining in contact with these surfaces. In addition to the asymmetric geometry of the tibial surfaces, trapezoidal joint laxity in flexion is also considered to be necessary for deep knee flexion. In the healthy knee, the lateral tibiofemoral joint gap is significantly lax [17]. Posterior displacement with posterior stress was larger in the lateral compartment at 90° and 135° flexion [18]. Although achieving normal stability and kinematics in replaced knees is challenging, these kinematic data should be beneficial for designing high-flexion total knee prostheses. 

This analysis has several drawbacks. First, the current approach to analyzing joint contact ignores the menisci, which are invisible on X-ray but obviously affect joint contact and load distribution. There is not currently an X-ray-based technique that will overcome this limitation, but 3D-to-2D registration techniques have the capability to reveal continuous dynamic *in vivo* kinematics. Second, the study included only five knees, but this cohort is similar to previous fluoroscopic studies that have analyzed four or five healthy knees [1, 2] and is consistent with minimizing X-ray exposure to healthy individuals while still obtaining important information. Finally, this study did not include the assessment of healthy patellofemoral kinematics, which also is important for designing knee replacements for natural flexion kinematics. 

## 4. Conclusions 

The lateral tibiofemoral contact translated posteriorly to the edge of the tibial surface at full flexion with 30° external and 5° valgus rotation relative to the tibia. The medial tibiofemoral contact remained centered on the tibial surface. The results of this study were generally consistent with reports from previous studies. Healthy knee kinematic data during full flexion may provide important insight for designing high-flexion total knee prostheses.
